# The Death and Life of the Resurrection Drug

**DOI:** 10.1371/journal.pntd.0002910

**Published:** 2014-07-10

**Authors:** Charles Ebikeme

**Affiliations:** Science Writer, Paris, France; New York University School of Medicine, United States of America

In his own words, it was the most dramatic moment of his scientific career. At the back of an auditorium in Nairobi, Kenya, Cyrus Bacchi met Simon Van Nieuwenhove. Bacchi, at the time, was essentially a biochemist whose interest was the African trypanosome. Van Nieuwenhove was a clinician who had worked for many years in the field in Africa. The topic of their conversation was eflornithine—a small and simple compound that would eventually make a big impact. Bacchi had shown that the compound had an effect on the parasite that causes Human African Trypanosomiasis. Van Nieuwenhove was dosing patients with it.

The story of eflornithine did not start with eflornithine.It started because no one knew how to purify an enzyme.

Bacchi was working on his thesis, which centred on the α-glycerophosphate shuttle in hemoflagellates. The shuttle acts as a way to transport reducing equivalents from the cytosol to the mitochondrion. α-glycerophosphate dehydrogenase was the enzyme he spent much of his time trying to purify. The reason he could not purify it was it was hidden within another compartment of the trypanosome. It would not be until several years later, and published in 1977, that Fred Opperdoes discovered the glycosome [1]: the organelle that encapsulates glycolysis in trypanosomes, and the organelle that was hiding the enzyme.

At every step of the purification process Bacchi lost activity in his enzyme extracts. Magnesium chloride, surprisingly, managed to boost activity. Bacchi looked into other nonmetallic compounds that would act as cations and eventually chose the polyamines: naturally occurring, nitrogen-containing cations whose concentration is closely controlled by the cell. Spermidine and spermine were found to be the best replacements for magnesium, yielding higher activities.

With evidence that the enzyme he was trying to purify was most likely locked within the glycosome, Bacchi moved his attention to how the polyamines were made within the trypanosome. This was an area that had amassed a lot of knowledge everywhere apart from in trypanosomes. It was in 1677 that Anton van Leeuwenhoek first observed spermatozoa in humans, dogs, and a host of other organisms, and he later discovered crystals of spermine phosphate in human semen. Modern research had identified the biologically active polyamines—spermidine and spermine—in plants and many types of mammalian cells. The biochemical pathways that make and degrade the polyamines, along with some of their enzymes, had also been identified.

At the time, nothing was known about polyamines in protozoa, and in trypanosomes in particular. Did these parasites contain polyamines? Could polyamine metabolism be a useful chemotherapeutic target?

## Eflornithine and Cancer

Cancer was always the initial target [2]–[6]. The rationale and scientific body of fact had been building for decades. During the 1970s, scientists at the Merrell Research Institute in Strasbourg began to synthesize inhibitors of a target enzyme they thought would lead to an eventual broad-spectrum cancer chemotherapy. The science had already shown a link between high levels of polyamines and rapid cell proliferation in prokaryotic and eukaryotic cells.

Rational drug design became increasingly popular as a method for drug development in the 1950s, achieving some sort of legitimate authority with the award of a Nobel Prize in 1988. That year, the prize in Physiology or Medicine was awarded jointly to Sir James W. Black, Gertrude B. Elion, and George H. Hitchings for their discoveries of “important principles for drug treatment.” Previous decades had seen drug development based on empirical chemical modification of natural products. The start of a more rational approach to drug design in the 1950s, with the emphasis on understanding basic biochemical and physiological processes as specific drug targets, was what eventually led to the development of eflornithine. What we had then was a new way of creating potential drugs. Eflornithine or D,L-alpha-difluoromethylornithine (DFMO) was the eventual result.

Eflornithine was one of a series of amino acid analogues whose design was based on a predicted enzymatic reaction, to generate mechanism-based inhibitors of amino acid decarboxylases at the active site of the enzyme. Ornithine decarboxylase (ODC) was the first enzyme in mammalian polyamine synthesis. Eflornithine was its suicide inhibitor—specific and irreversible. Inhibition of ornithine decarboxylase proved it to be a key enzyme involved in polyamine biosynthesis, resulting in a consequent impairment of cellular division, or—in the case it was intended for—cancer's hyperproliferation.

Great was the promise of eflornithine [7]. Its development spurred on a large range of clinical trials. The early days of eflornithine as a cancer treatment quickly came to a halt as the many side effects came to the forefront [8]. High doses for prolonged periods caused diarrhoea, abdominal pain, and emesis, as well as moderate anaemia, leukopenia, and thrombocytopenia. Soon, the reality in study after study was that it would fail to be of use as an antitumour agent.

## Meetings and Mice

In 1977, Bacchi attended the Polyamine Gordon Conference in New Hampshire, where research on eflornithine was initially presented. His first question was how he could obtain it for use in his research. Eflornithine was scarce. Initially, he managed to obtain only 25 milligrams, not enough to do what he really wanted with it: treat trypanosome-infected mice with 2% eflornithine in drinking water. Two years later he would attend the same Gordon Conference. This time he met the right person—Peter McCann, who was Liaison for pharmaceutical–university relations at Merrell Pharmaceuticals in Cincinnati, the company that had been developing eflornithine as an anticancer agent. Soon after that meeting, McCann sent Bacchi 25 grams.

The original paper published in the journal *Science* in 1980, entitled “Polyamine Metabolism: A Potential Therapeutic Target in Trypanosomes,” lists among the authors Dr. Al Sjoerdsma [9]. At the time he was the Director of the Merrell Research Institute. The collaboration was born when he gave the initial okay to send such a significant amount of eflornithine to Bacchi. Without Al Sjoerdsma, there would be no story of eflornithine.

On receipt of the 25 grams of eflornithine, Cyrus and his colleague Henry Nathan immediately began dosing trypanosome-infected mice with eflornithine at a 2% solution of eflornithine in their drinking water. Every scientist is always hopeful before an experiment, but not knowing if the dose or the time would be enough to allow eflornithine to work, or if eflornithine itself would affect the mice, there was every reason to be doubtful.

The mice survived.

Even before their article detailing eflornithine's curative effects on mice was published in *Science* in 1980, the compound was already on its way to Geneva and the World Health Organization (WHO).

## Conference Cures

At the Kenyatta International Conference Centre, Bacchi and Van Nieuwenhove talked eflornithine for an hour. It was at the International Conference of Protozoology, just four short years after Bacchi had published the paper in *Science* detailing how the small amino acid–like compound could stop the invariably fatal outcome of sleeping sickness. It worked on mice, showing curative properties that were described as miraculous. Van Nieuwenhove had actually tried it on people. Van Nieuwenhove had tested it on patients with significant success using a six to eight week oral treatment regimen.

Van Nieuwenhove had been working in southern Sudan for a long time on a sleeping sickness control project run by the Belgian government. Those present at the time have recounted how the very first trials of eflornithine took place with very little planning involved. Van Nieuwenhove landed in Sudan from Geneva with eflornithine in his suitcase and with just a basic idea about how to treat patients with it.

Twenty patients, 18 of whom had late-stage trypanosomiasis, were given eflornithine by mouth for up to six weeks. The drug was diluted in fruit juice, at 400 milligrams per kilogram per day—four times a day. And despite problems with diarrhoea, ototoxicity, and hair loss, eflornithine worked. It cured most who took it, even those with the more serious late-stage disease.

## Resurrecting Patients

In the space of two years, trypanosomiasis was diagnosed in five patients at three different Belgian hospitals [10]. All had recently lived in endemic parts of the country formerly known as Zaire. Two patients had early-stage disease, and three manifested with late-stage disease. Henri Taelman was a doctor in Antwerp during the 1980s. In March of 1983, a 28-year-old woman was admitted to his hospital with complaints of “nocturnal itching” that had lasted for several months. But it was in a hospital in Brussels that the most dramatic case was to be found.

In January of 1983, a 55-year-old woman from Zaire came back to life from a comatose state. The woman had moved to Belgium three years earlier to be with her children. After weeks of searching, trypanosomes were detected in a bone marrow smear. By that time, melarsoprol could not be given as she was in a very bad state. Her doctors described her condition as terminal. Then, her doctors decided to try eflornithine.

After just 24 hours of treatment, there were no longer parasites detected in her blood, and on the third day she awoke from her coma. After a week, she was fully conscious. Without the option to treat with melarsoprol, this new drug eflornithine was the only choice. Eflornithine would later go on to be referred to as the “resurrection drug” for its Christ-like ability to bring back those who were seemingly dead.

## Arsenic and Old Drugs

To understand the history of eflornithine is to understand arsenic. We think of arsenic and we think of poison, and we think of old places with decaying concrete and arsenical pigment in the wallpapers [11]. The frontline treatment for trypanosomiasis was arsenic, in the form of the drug Melarsoprol [12].

Melarsoprol is a dangerous drug. How could a cocktail of arsenic and antifreeze be anything less? Melarsoprol, a remnant from a time when the once-referred-to-as “third world” had been somewhat of a laboratory testing ground for the West, is a drug that would never achieve licensing in this day and age.

It was in 1858 that the famed Scottish missionary and explorer David Livingstone suggested using a solution of arsenic to treat sleeping sickness. Over the years, many versions of arsenic were used to treat sleeping sickness [13]. In 1949, melarsoprol came and stayed, as it was—ironically—the safer alternative to all the other arsenic-based treatments. It causes a reactive encephalopathy in roughly 10% of patients, with an overall mortality rate of 2%–5%—unacceptable by today's pharmaceutical standards [14]. For a long time, melarsoprol was still the only effective drug against second-stage sleeping sickness, and because eflornithine is not effective against *Trypanosoma brucei rhodesiense* (which accounts for 2% of all reported cases), melarsoprol will remain for a long time to come. It has to be administered under direct medical supervision in a hospital, intravenously, from plastic tubes that won't melt from the arsenic.

Towards the end of the last century, something odd started to happen. Patients were no longer responding to treatments of melarsoprol [15]. Death rates threatened to spiral again, much like they had done before, during most of the 1960s. A growing number of cases of trypanosomiasis were not responding to the old drugs available. Treatment failures reached alarming levels. Up to 30% had been documented in some foci across Angola, the Democratic Republic of Congo, southern Sudan, and Uganda.

Eflornithine was needed more than ever.

## Death and Vanity

When Bacchi met Van Nieuwenhove at the back of that auditorium in the Kenyatta Centre, it was an exchange of expertise. Van Nieuwenhove wanted to know how eflornithine acted and Bacchi wanted to hear about the clinical experiences. This was at a time when eflornithine was still young and in their hands – later to enter a different kind of world.

Poverty has always been a hurdle in global health. The financial disincentive for new drug development for afflictions of the poor is always front and centre. Only in recent decades have models come about to tackle the problem of simple market economics. The fact is in the numbers—most of the drugs currently used to treat this disease were developed more than half a century ago.

Eflornithine had a life that lasted only nine years [16]. When it was licensed and approved for treatment for trypanosomiasis in 1990 it seemed like a solution—less toxic than melarsoprol [17]. But for eflornithine, production would stop in 1999.

The 1990s started a trend that has only escalated since. A whole host of pharmaceutical companies merged and consolidated their efforts. In 1989, Merrell Dow merged with Marion Laboratories to be known as Marion Merrell Dow. Gone were the large portfolios of drugs, to make way for fewer, more profitable therapeutics. The 1990s saw the wave of lifestyle drugs reach its peak.

Marion Merrell Dow, after the merger, was granted marketing approval and orphan drug status for eflornithine in 1990 by the United States Food and Drug Administration (FDA), under the trade name Ornidyl—becoming the first new drug in more than 40 years for the treatment of African trypanosomiasis.

Eflornithine's end came as Sanofi (who had by then acquired Marion Merrell Dow) gave up on its production, seeing it as too costly. Final stockpiles were sold off to Médecin Sans Frontières and patent rights given to the WHO in order to find another manufacturer. For the longest of times, no other manufacturer could be found.

If it was not for vanity, eflornithine would be lost to medical history. Its rebirth came in the form of a face cream. In 2000, eflornithine was given a new lease of life when it was approved by the FDA for use as a topical cream to control the growth of facial hair. Vaniqa was the name of the marketed cream, and eflornithine was its active ingredient [16].

In February of 2000, the CBS News program “60 Minutes” documented the fate of late-stage sleeping sickness patients dying for lack of treatment or enduring painful intravenous therapy with Melarsoprol. They followed up by showing the Vaniqua advertisement. The response was overwhelming and the pharmaceutical industry was held up to a higher morality standard—condemned for withholding medicine from the Third World.

It was thought of as “obscene” at the time. The drug that had been waking the diseased up from comas in Congo, Sudan, and much of Africa was seen in a six-page ad in Cosmopolitan magazine. At the time, it was a scandal. Looking back, it was a scandal that probably saved the lives of millions.

In the wake of the scandal, Bristol Myers Squibb agreed to donate the drug free of charge. And then in 2001, Aventis agreed to continue to produce eflornithine.

## Secret to Success

During the 1960s, trypanosomiasis was, without a doubt, at its lowest level since records began. When a joint expert committee met in Geneva in late June of 1962, the published report hailed “spectacular successes” where control efforts had reduced the incidence of the disease to extremely low levels. Old-world colonial territories like Rhodesia and Nyasaland reported limited numbers of new cases every year. Double-digit figures of new cases were not exceptional for many parts of the continent. For the next few decades, it would seem that sleeping sickness would no longer pose a threat to human health and development on the African continent.

Today, eflornithine exists in combination therapy with nifurtimox [18]–[24], another drug that discovered a new lease of life against another disease (registered for use against American trypanosomiasis but not for human African trypanosomiasis). By 2050, trypanosomiasis will no longer be a problem. That is the hope, and that is what the WHO asserts it is on track to accomplishing. Eradication and elimination are the next great hope for a disease where thoughts of a vaccine seem like fanciful flights of science fiction. It seems the time to develop new drugs is long gone—or, at least, that time is now or never.

Free donations of eflornithine, melarsoprol, pentamidine, suramin, and nifurtimox by pharmaceutical companies over the years have helped in stemming the tide during much of the last hundred years. The past decades have seen the abandonment of “business-as-usual” tactics by many of the large drug companies—quick to embrace a much-needed openness about intellectual property and access to medicines. But simple economics remain. As those that suffer from these afflictions dwindle in their numbers, so will the impetus to do something about it. Few pharmaceutical plants are geared to manufacture small amounts of drug to cater for ever-decreasing numbers of people. The difference is between kilos and metric tonnes.

Eventually, eflornithine will be reconciled to medical history once again as better and more efficacious drugs are developed and other modes to combat the disease are put into practice. Perhaps, it will find use once again in another way, against another disease.
